# Development and validation of a nomogram for predicting suicide risk factors in thyroid cancer patients following diagnosis: a population-based retrospective study

**DOI:** 10.3389/fendo.2025.1392283

**Published:** 2025-09-30

**Authors:** Jie Zhou, Mengjie Tian, Xiangchen Zhang, Lingyi Xiong, Jinlong Huang, Mengfan Xu, Xinjun Liang, Shaozhong Wei

**Affiliations:** ^1^ Department of Gastrointestinal Oncology Surgery, Hubei Cancer Hospital, Tongji Medical College, Huazhong University of Science and Technology, Wuhan, Hubei, China; ^2^ Colorectal Cancer Clinical Research Center of Hubei Province, Wuhan, Hubei, China; ^3^ Colorectal Cancer Clinical Research Center of Wuhan, Wuhan, Hubei, China; ^4^ Department of Abdominal Oncology, Hubei Cancer Hospital, Tongji Medical College, Huazhong University of Science and Technology, Wuhan, Hubei, China

**Keywords:** suicide, thyroid cancer, nomogram, predictors, SEER, population-based study

## Abstract

**Objectives:**

To develop and validate a user-oriented nomogram of suicide risk in thyroid cancer patients to enable clinicians to identify and intervene in a timely manner with high-risk subgroups.

**Methods:**

This was a retrospective, population-based cohort study in which patients with thyroid cancer diagnosed from the Surveillance, Epidemiology, and End Results (SEER) database between 2000 and 2020 were include. Optimized features were screened by the least absolute shrinkage and selection operator (LASSO) regression model. Subsequently, we selected variables with nonzero coefficients, entered them into a Cox proportional hazards regression model and constructed a visualized nomogram model predicting suicide. We implemented receiver operating characteristic curves (ROC), calibration curves, decision curve analysis (DCA), and internal validation to assess the discrimination, calibration, clinical applicability, and generalizability of the nomogram.

**Results:**

To our knowledge, this is the first nomogram specifically designed for thyroid cancer patients, integrating histopathological, therapeutic, and socioeconomic predictors. Furthermore, the calibration curves for this nomogram fit well with the diagonal, and the C-indexes for the training and testing sets were 0.760 and 0.724, respectively, and the decision curve analysis indicated clinical benefit.

**Conclusion:**

This study successfully identified risk factors for suicide in patients with thyroid cancer and developed a nomogram that provides patients with an individualized, quantifiable assessment of suicide risk and assists clinicians in identifying and intervening in potential suicides.

## 1 Introduction

With advances in diagnostic imaging and surveillance, the detection rate of thyroid cancer continues to increase. According to the International Agency for Research on Cancer of the World Health Organization, the incidence of thyroid cancer is the ninth highest in the world (1, 2). In addition, thyroid cancer is the most common malignant tumor among women in the United States (3). Thyroid cancer patients have a cumbersome financial burden, and the bankruptcy rate among survivors is higher than for other cancer types in America (4).

Suicide, the tenth leading cause of death in North America, confronts us with a great public health challenge (5). The last decade has seen the development of novel molecular-based individualized systematic management, leading to an improving prognosis for thyroid cancer (6). Despite a favorable prognosis, the risk of suicide in thyroid cancer patients is 5.3 times higher than in the general population (95% CI 3.8-7.1), and this “survival-psychological paradox” constitutes a central research gap (7). A meta-analysis based on the SEER database showed that the standardized suicide mortality rate (SMR=8.6) was significantly higher in thyroid cancer patients than in solid tumors such as breast cancer (SMR=1.9) (8). A multi-layered connection exists between thyroid cancer and elevated suicide risk. Biologically, thyroid hormone dysregulation directly affects mood regulation centers, particularly through hypothalamic-pituitary-thyroid axis modulation of serotonin systems (9). Psychosocially, the misperception of thyroid cancer as a “good cancer” leads to systemic minimization of patient distress, creating a unique “diagnosis-expectation disparity” trauma (10). Once upon a time, prolonging survival time was the main goal of cancer treatment, sometimes even at the expense of the patient’s quality of life and psychological well-being. The long series of cancer diagnosis, treatment, and recovery brings great emotional trauma to the patient, which in turn affects their ability to fight cancer. Some scholars argue that suicides caused by difficult or hopeless situations, such as terminal illness, are socially and legally recognizable and can be considered a rational as well as dignified behavior (11).

There is little literature that focuses on suicide in thyroid cancer patients singularly rather than as part of head and neck cancer. Much of the past research has been invested more in head and neck cancer suicides (8, 12, 13), possibly because its treatment modalities tend to cause disfigurement and impairments in swallowing, breathing, and speech (14). Despite the generally excellent prognosis of thyroid cancer, survivors’ quality of life of is troubling (15). The quality of life in patients with differentiated thyroid cancer (DTC) is worse than that of the general population and, surprisingly, even worse than that of survivors of other colorectal, breast, and malignant glioma types with lower survival rates and more aggressive treatments (16). A survey of suicide mortality among U.S. pediatric cancer survivors noted the highest rate of 39.8 per 100,000 person-years among thyroid cancer patients (17). Surgery, the cornerstone of thyroid cancer treatment, has two major complications: recurrent laryngeal nerve injury and permanent hypoparathyroidism, which are major risk factors for anxiety in thyroid cancer patients (18). Some patients are overly concerned about the unsightliness associated with horizontal neck incisions, which affects their quality of life (19) and even suicide (20). Secondly, TSH suppression by high-dose thyroxine replacement postoperatively is associated with worse energy status and immense fatigue (21). In addition, among patients treated with radioiodine, those with doses greater than 150 mCi tended to have more severe pain, swallowing, chewing, speech, taste, anxiety, and mixed disorders (22). Furthermore, the characteristics of younger age at thyroid cancer onset and good survival rates increase the risk of subsequent second tumors (23), which is part of the rationale for its high suicide rate. Therefore, more attention should be paid to the risk management of suicide deaths in patients with thyroid cancer.

Therefore, to identify high-risk subgroups of patients with thyroid cancer who die by suicide, we executed a population-based cohort study. This study is expected to provide a scientific basis for the development of specific suicide death management strategies for patients with thyroid cancer, thereby reducing distress, anxiety and suicide risk in survivors.

## 2 Methods

### 2.1 Data source

Thyroid cancer cases diagnosed during 2000-2020 were selected from the Surveillance, Epidemiology, and End Results (SEER) database. The database is structured by National Cancer Institute (NCI) and contains information from 17 population-based registries (SEER-17; Alaska, Connecticut, Atlanta, Greater Georgia, Rural Georgia, San Francisco-Oakland, San Jose-Monterey, Greater California, Hawaii, Iowa, Kentucky, Los Angeles, Louisiana, New Mexico, New Jersey, Seattle-Puget Sound, and Utah). The SEER-17 database covers approximately 26.5% of the U.S. population and racially representative, which contains patient demographics (gender, age, race, marital status), tumor diagnostic information (stage, grading, histological type, tumor size), treatment and follow-up data. No Institutional Review Board approval was required for the Hubei Cancer Hospital since this study involved neither interaction with human participants nor the use of any personally identifiable information.

### 2.2 Study population

Cases of thyroid cancer diagnosed during the period 2000-2020 were included, which were categorized according to histological type (*International Classification of Diseases in Oncology, Third Edition*; topography code C73): papillary thyroid cancer (histological codes 8050, 8260, 8340-8344, 8350, 8450-8460), follicular thyroid cancer (8290, 8330-8335), medullary thyroid carcinoma (8345, 8510-8513), anaplastic thyroid cancer (8000, 8012, 8020-8022, 8030-8032, 8337) and others. Only microscopically confirmed cases and the corresponding first record were selected, while cases identified only from autopsy records or death certificates were excluded. We extracted patient demographic data (age, sex, race, marital status, annual household income, place of residence), tumor-related details (histologic type, surgery, radiation therapy, chemotherapy), and follow-up information (survival status, cause of death, and time to survival) through the SEER*Stat Version 8.4.2.0.

Reasons for death were assigned on the basis of the death certificate documented and confirmed by the attending physician, with deaths due to “suicide and self-inflicted injury” following a diagnosis of thyroid cancer being the main outcome. Survival time is measured in months, with a minimum non-zero value of 1 month. Marital status was portrayed as married/cohabiting, divorced/separated/windowed and single (never married). We define place of residence as metropolitan (population greater than or equal to 1 million), middle urban (population greater than or equal to 250 thousand), small urban (population less than 250 thousand), and rural area based on the urban-rural continuum code provided by the SEER database.

### 2.3 Statistical methods

Data were randomly and non-returned drawn into training and testing cohorts in a ratio of 7:3. Predictor screening and model construction were performed in the training cohort, while model evaluation was accomplished in the testing cohort. Student’s t-test was used to analyze continuous variables while chi-square or Fisher’s exact test was used to compare categorical variables. R software 4.2.2 (R Foundation for Statistical Computing, Vienna, Austria) was deployed for all statistical analyses. Statistical significance was evaluated at the alpha level of P< 0.05, and all assumptions were two-sided.

To construct the final predictive model, we employed the least absolute shrinkage and selection operator (LASSO) combined with Cox proportional hazards regression to identify significant suicide risk factors. As a regularized regression method, LASSO regression not only performs well in screening variables and creating simple models, but is also more effective in solving overfitting problems (24). LASSO regression is highly suitable for models with high degree of multicollinearity and it helps in variable selection and parameter elimination by shrinking the values towards the center point. In addition, it adds a penalty value equal to the absolute value of the coefficients, and variables with zero coefficients will eventually be dropped from the model (25). Cox proportional hazards model is suitable for analyzing survival data over time (26), and it can be used to adjust for baseline differences between groups and provide hazard ratios (calculation of confidence intervals by 1000 iterations) to quantify the effect of any single factor on the survival curve (27, 28).

The discriminative efficacy and generalization ability of the model for the LASSO-Cox model was evaluated by Harrell’s concordance index (C-index). We next plotted calibration curves using the Bootstrap method of resampling 1000 times to assess the agreement between observed and predicted probabilities. The total score of each patient in the nomogram was extracted, and the ROC curve was used to obtain the best cutoff value. Based on this cutoff value, all patients were categorized into high-risk and low-risk groups, and Kaplan-Meier (K-M) curves were used to describe the survival curves for suicide in the two groups, and the differences between the groups were compared using the log-rank test. In addition, decision curve analysis (DCA) quantified the net benefit at different probability thresholds to assess clinical applicability.

## 3 Results

### 3.1 Characteristics of patients

Of the 213,932 thyroid cancer cases diagnosed among residents of SEER-17 region during 2000-2020, 199,135 (93.1%) were finally included in this study (**Table 1**). Females (149,825 [75.2%]) and white patients (163,086 [81.9%]) comprised the majority of cases. The mean (± standard deviation) age at diagnosis was 50.2 (± 15.9) years. The most common histological types were PTC (87.4%) and FTC (7.7%). The other features selected for inclusion are shown in **Table 1**. During the period 2000-2020, 169 of the eligible cases died by suicide, of which 51.5% were male and 89.9% were white. The correlation between patients who survived, died by suicide, and those who died by non-suicide and baseline information is shown in **Table 2**, with significant differences between the three in terms of age, gender, race, marital status, histological type, surgery, annual household income, place of residence, year of diagnosis, and duration of follow-up.

**Table 1:** Baseline clinicopathological characteristics of patients in training and testing cohorts (N=199,135).

Variables	Total	Training cohort (N=139394)		Testing cohort (N=59741)		P-value
		N	%	N	%	
Age						0.505
<38	46725	32641	23.4%	14084	23.6%	
38∼49	50254	35100	25.2%	15154	25.4%	
50∼61	51624	36166	25.9%	15458	25.9%	
≥ 62	50532	35487	25.5%	15045	25.2%	
Gender						0.897
Male	49310	34505	24.8%	14805	24.8%	
Female	149825	104889	75.2%	44936	75.2%	
Race						0.226
White	163086	114233	81.9%	48853	81.8%	
Black	12838	8900	6.38%	3938	6.59%	
Others^a^	23211	16261	11.7%	6950	11.6%	
Marital Status						0.708
Married/Cohabitation	127607	89402	64.1%	38205	64.0%	
Separated/Divorced/Windowed	28044	19616	14.1%	8428	14.1%	
Single (never married)	43484	30376	21.8%	13108	21.9%	
Surgery						0.450
Performed	190760	133500	95.8%	57260	95.8%	
Not performed	8375	5894	4.23%	2481	4.15%	
Radiotherapy						0.327
Performed	88712	61947	44.4%	26765	44.8%	
No/Unknown	109502	76799	55.1%	32703	54.7%	
Refused	921	648	0.46%	273	0.46%	
Chemotherapy						0.216
Performed	2278	1622	1.16%	656	1.10%	
No/Unknown	196857	137772	98.8%	59085	98.9%	
Histological type						0.366
Papillary	174033	121738	87.3%	52295	87.5%	
Follicular	15256	10679	7.66%	4577	7.66%	
Medullary	3568	2511	1.80%	1057	1.77%	
Anaplastic	3149	2241	1.61%	908	1.52%	
Other	3129	2225	1.60%	904	1.51%	
Annual household income						0.590
<$45,000	8611	6047	4.34%	2564	4.29%	
$45,000 - $64,999	47204	33117	23.8%	14087	23.6%	
≥$65,000	143320	100230	71.9%	43090	72.1%	
Residence						0.946
Metropolitan	120577	84355	60.5%	36222	60.6%	
Middle urban	44334	31064	22.3%	13270	22.2%	
Small urban	14658	10253	7.36%	4405	7.37%	
Rural area	19566	13722	9.84%	5844	9.78%	
Year of diagnosis						0.047
2000-2010	85261	59481	42.7%	25780	43.2%	
2011-2020	113874	79913	57.3%	33961	56.8%	
Follow-up time						0.242
<39	49394	34638	24.8%	14756	24.7%	
39-86	49518	34731	24.9%	14787	24.8%	
87-145	50363	35304	25.3%	15059	25.2%	
≥146	49860	34721	24.9%	15139	25.3%	

a Other race includes those who were Asian or Pacific Islander or American Indian or Alaska Native.

**Table 2:** Relationship between the survival status and clinicopathological characteristics.

Variables	Non-suicide death		Suicide		Alive		P-value
Cases	24567		169		174399		
Age							<0.001
<38	786	3.20%	33	19.5%	45906	26.3%	
38∼49	2273	9.25%	47	27.8%	47934	27.5%	
50∼61	5198	21.2%	45	26.6%	46381	26.6%	
≥62	16310	66.4%	44	26.0%	34178	19.6%	
Gender							<0.001
Male	9704	39.5%	87	51.5%	39519	22.7%	
Female	14863	60.5%	82	48.5%	134880	77.3%	
Race							<0.001
White	20149	82.0%	152	89.9%	142785	81.9%	
Black	2009	8.18%	4	2.37%	10825	6.21%	
Others^a^	2409	9.81%	13	7.69%	20789	11.9%	
Marital Status							<0.001
Married/Cohabitation	13715	55.8%	90	53.3%	113802	65.3%	
Separated/Divorced/Windowed	7209	29.3%	31	18.3%	20804	11.9%	
Single (never married)	3643	14.8%	48	28.4%	39793	22.8%	
Surgery							<0.001
Performed	19922	81.1%	160	94.7%	170678	97.9%	
Not performed	4645	18.9%	9	5.33%	3721	2.13%	
Radiotherapy							–
Performed	10629	43.3%	73	43.2%	78010	44.7%	
No/Unknown	13758	56.0%	93	55.0%	95651	54.8%	
Refused	180	0.73%	3	1.78%	738	0.42%	
Chemotherapy							–
Performed	1550	6.31%	5	2.96%	723	0.41%	
No/Unknown	23017	93.7%	164	97.0%	173676	99.6%	
Histological type							–
Papillary	17141	69.8%	148	87.6%	156744	89.9%	
Follicular	2848	11.6%	10	5.92%	12398	7.11%	
Medullary	935	3.81%	2	1.18%	2631	1.51%	
Anaplastic	2340	9.52%	6	3.55%	803	0.46%	
Other	1303	5.30%	3	1.78%	1823	1.05%	
Annual household income							<0.001
<$45,000	1341	5.46%	4	2.37%	7266	4.17%	
$45,000 - $64,999	6600	26.9%	50	29.6%	40554	23.3%	
≥$65,000	16626	67.7%	115	68.0%	126579	72.6%	
Residence							<0.001
Metropolitan	14058	57.2%	90	53.3%	106429	61.0%	
Middle urban	5190	21.1%	41	24.3%	39103	22.4%	
Small urban	2094	8.52%	20	11.8%	12544	7.19%	
Rural area	3225	13.1%	18	10.7%	16323	9.36%	
Year of diagnosis							<0.001
2000-2010	16281	66.3%	111	65.7%	68869	39.5%	
2011-2020	8286	33.7%	58	34.3%	105530	60.5%	
Follow-up time							<0.001
<39	10314	42.0%	64	37.9%	39016	22.4%	
39-86	6143	25.0%	48	28.4%	43327	24.8%	
87-145	5080	20.7%	32	28.4%	45251	25.9%	
≥146	3030	12.3%	25	14.8%	46805	26.8%	

a Other race includes those who were Asian or Pacific Islander or American Indian or Alaska Native.

### 3.2 Prediction model built based on Lasso-Cox regression

**Figure 1:**
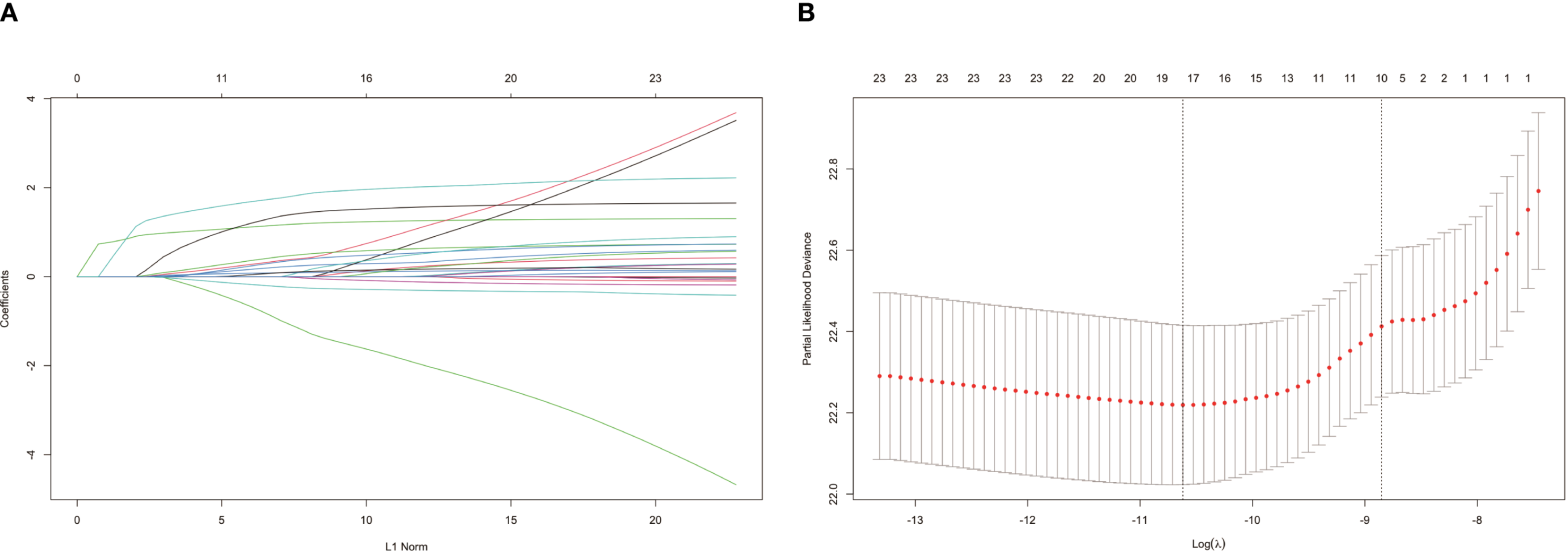
Filtrating prediction factors using the least absolute shrinkage and selection operator (LASSO) regression. **(A)** LASSO coefficient path diagram for risk factors; **(B)** Cross validation curve.

We used LASSO regression model to screen for risk factors, and the variation in the coefficients of these variables is characterized in **Figure 1A**. Applying the 10-fold cross-validation method to the iterative analysis, we compared the model with less than one standard error and fewer variables with the minimum error to obtain the best lambda, corresponding to the model with superior performance but the smallest number of variables (**Figure 1B**). The selected 11 features with non-zero coefficients include age, gender, race, marital status, surgery, radiation therapy, chemotherapy, histological type, annual family income, and place of residence.

**Figure 2:**
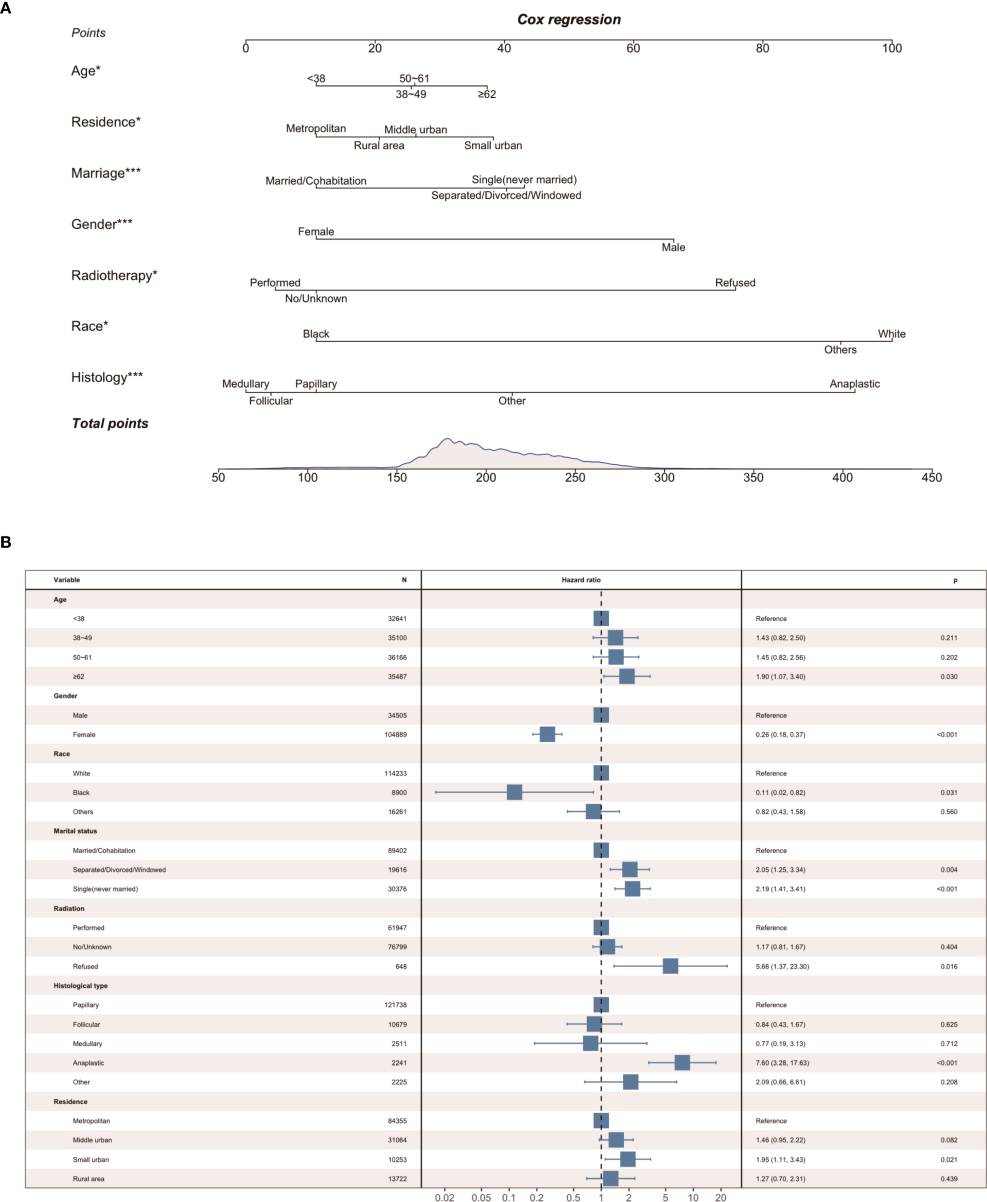
**(A)** Proposed nomogram for suicidality; **(B)** Forest plot of the model.

Parameters screened based on LASSO regression were incorporated into the next univariate and multivariate Cox proportional hazards regression models. As shown in **Table 3**, we identified the seven best predictors, including age at diagnosis, place of residence, marital status, gender, radiotherapy, race, and histological type. Subsequently, we constructed a nomogram based on these results (**Figure 2A**), and the forest plot of the model is shown in **Figure 2B**.

**Table 3:** Univariate and multivariate analyses of suicide risk in the training cohort (N=139394).

Variables	Univariate			Multivariate		
	HR	95%CI	P-value	HR	95%CI	P-value
Age						
<38	Reference			Reference		
38∼49	1.24	0.72-2.13	0.4338	1.42	0.81-2.49	0.2153
50∼61	1.38	0.81-2.36	0.2363	1.43	0.81-2.53	0.2166
≥62	2.14	1.27-3.62	0.0043	1.88	1.05-3.36	0.0333
Gender						
Male	Reference			Reference		
Female	0.26	0.18-0.37	<0.0001	0.26	0.18-0.38	<0.0001
Race						
White	Reference			Reference		
Black	0.12	0.02-0.87	0.0360	0.11	0.02-0.82	0.0314
Others^a^	0.67	0.35-1.28	0.2275	0.83	0.43-1.58	0.5645
Marital Status						
Married/Cohabitation	Reference			Reference		
Separated/Divorced/Windowed	1.81	1.13-2.91	0.0142	2.06	1.26-3.36	0.0038
Single (never married)	1.6	1.06-2.43	0.0253	2.20	1.41-3.42	0.0005
Surgery						
Performed	Reference					
Not performed	1.55	0.57-4.21	0.3866			
Radiotherapy						
Performed	Reference			Reference		
No/Unknown	1.11	0.78-1.59	0.5517	1.20	0.83-1.73	0.3275
Refused	5.61	1.37-23.01	0.0166	5.91	1.43-24.33	0.0139
Chemotherapy						
Performed	Reference			Reference		
No/Unknown	0.14	0.05-0.37	0.0001	0.35	0.11-1.12	0.0771
Histological type						
Papillary	Reference			Reference		
Follicular	0.94	0.47-1.85	0.8493	0.84	0.43-1.67	0.6259
Medullary	1.05	0.26-4.27	0.9421	0.72	0.18-2.96	0.6518
Anaplastic	9.42	4.12-21.56	<0.0001	5.63	2.16-14.7	0.0004
Other	2.31	0.73-7.28	0.1530	1.98	0.63-6.29	0.2448
Annual household income						
<$45,000	Reference					
$45,000 - $64,999	6.5	0.89-47.32	0.0646			
≥$65,000	4.33	0.6-31.14	0.1451			
Residence						
Metropolitan	Reference			Reference		
Middle urban	1.44	0.94-2.19	0.0927	1.46	0.96-2.23	0.0803
Small urban	1.93	1.1-3.4	0.0219	1.95	1.11-3.44	0.0207
Rural area	1.3	0.71-2.36	0.3950	1.27	0.7-2.31	0.4386

a Other race includes those who were Asian or Pacific Islander or American Indian or Alaska Native.

### 3.3 Performance and internal validation of the nomogram

**Figure 3:**
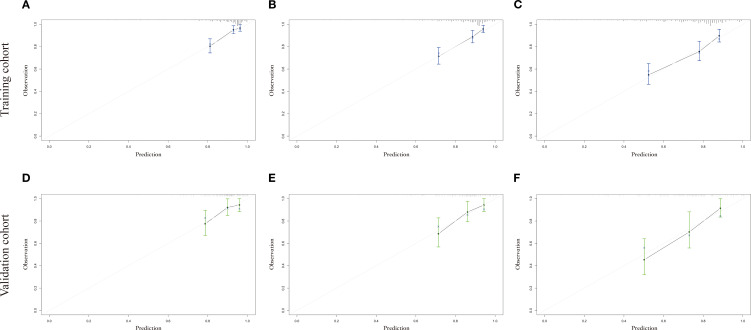
The calibration plot for prediction in the training and testing cohorts. **(A-C)** shows the 3-year, 5-year and 10-year suicide survival endpoints in the training cohort, and **(D-F)** shows the 3-year, 5-year and 10-year suicide survival endpoints in the testing cohort.

**Figure 4:**
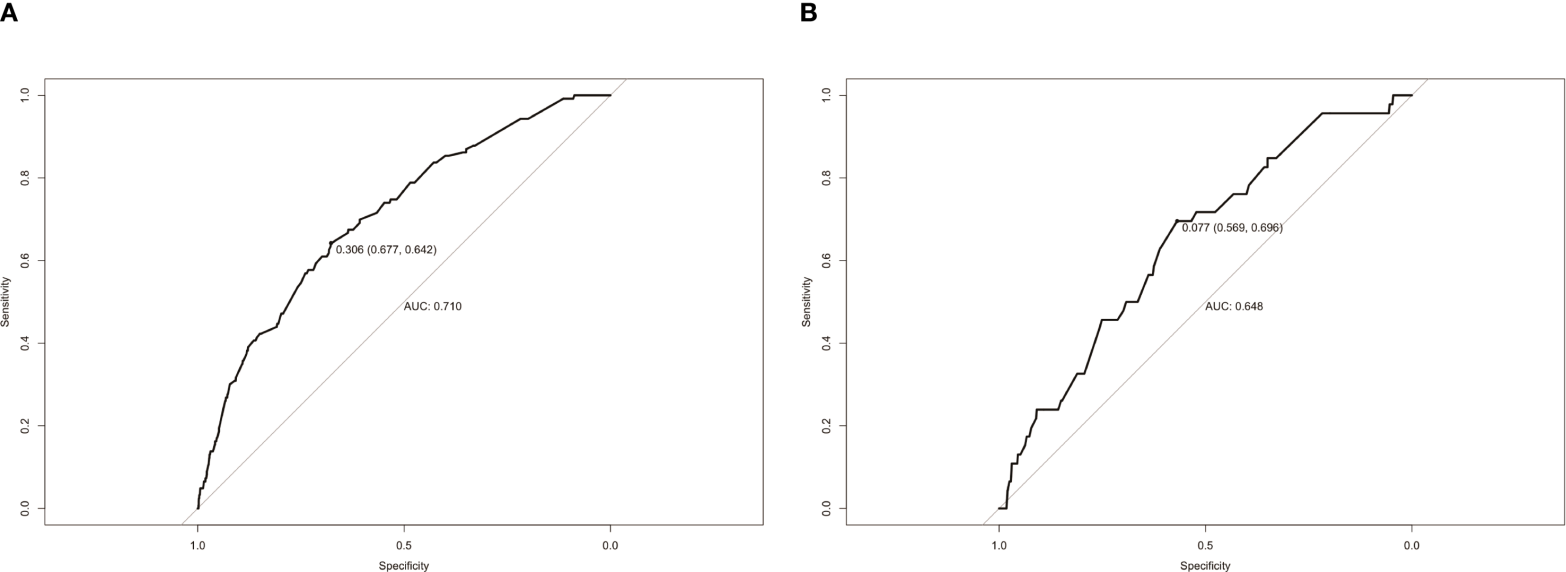
ROC curves of the study’s generated nomogram in the **(A)** training cohort; **(B)** testing cohort.

The calibration curves used to predict the nomogram for suicide risk in thyroid cancer patients showed good agreement in both the training and testing cohorts (**Figure 3**). The C-index for the predicted nomogram was 0.760 (95% CI: 0.717-0.802) in the training cohort and 0.724 (95% CI: 0.644-0.803) in the testing cohort, suggesting a superior discriminative capability of the model. The sensitivity and specificity of the training set were 0.677 and 0.642, respectively, with an optimal cut-off of 0.306 (**Figure 4**).

### 3.4 Clinical application

**Figure 5:**
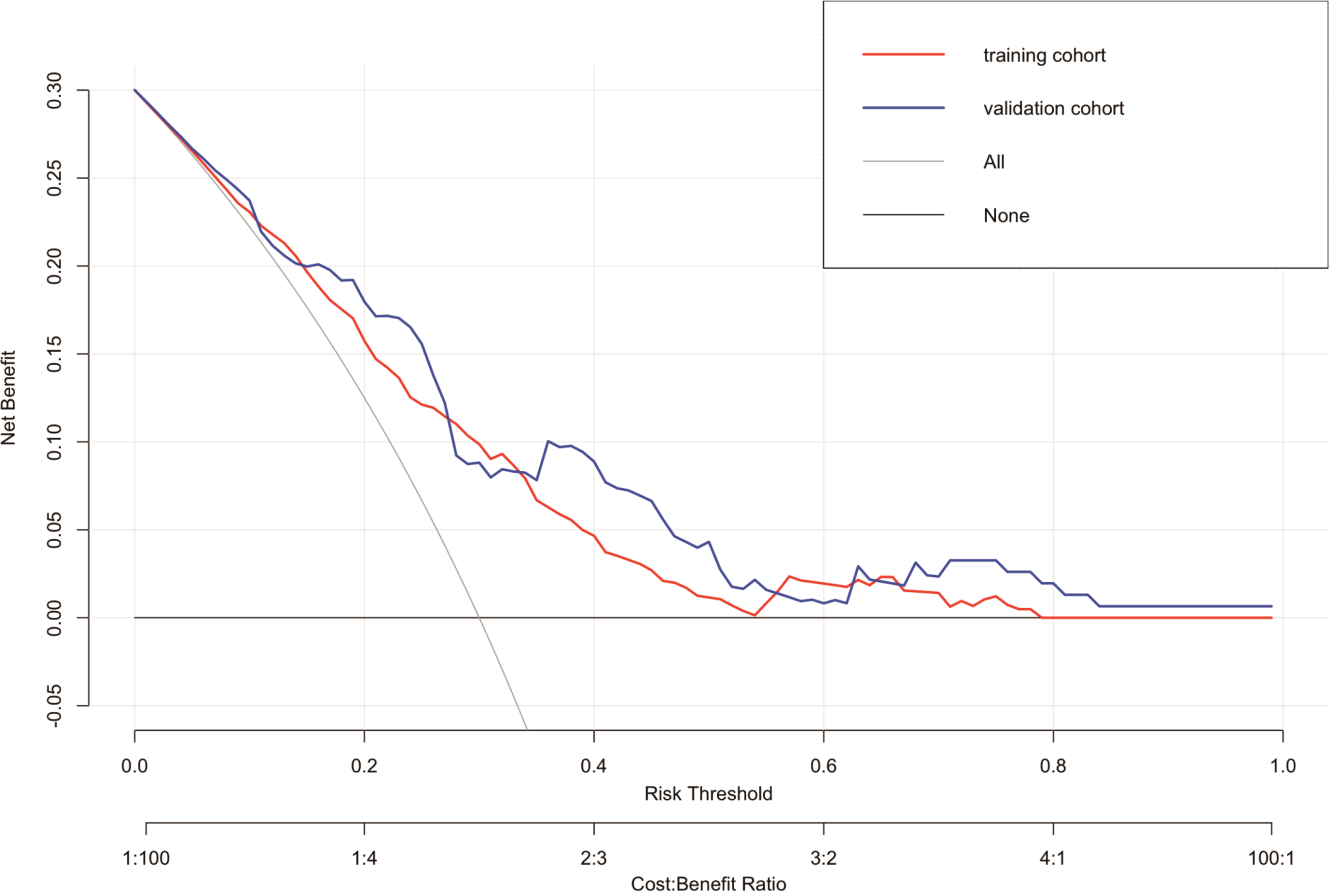
Decision curve analysis (DCA) for the nomogram.

**Figure 6:**
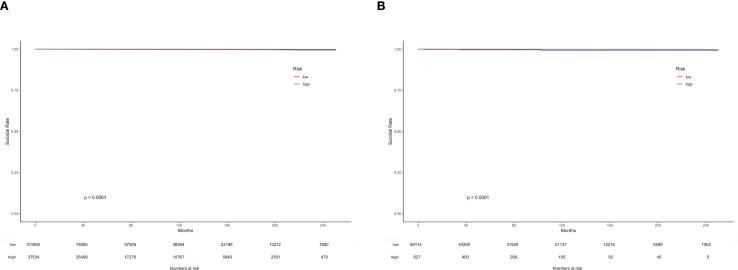
K-M curves of TC patients in the training cohort **(A)** and testing cohort **(B)** according to risk grouping.

In the suicide nomogram, the decision curve analysis (DCA) is shown in **Figure 5**. The decision curves for both the training and testing cohorts demonstrate that using the predictive model to predict suicide risk in thyroid cancer patients may add additional clinical benefit if the probability threshold for suicide is not less than 5%. That is, when the threshold for the incidence of suicide is not less than 5%, using the model to predict suicide risk is more beneficial than intervening or not intervening for all patients. Meanwhile, patients were categorized into low-risk (total score < 173.934) and high-risk groups (total score≥ 173.934) based on the best cutoff value of the total risk score in the nomogram. The K-M curves for both the training and testing sets showed that there was a significant difference in suicide survival between the different risk groups (**Figures 6A, B**).

## 4 Discussion

Differentiated thyroid cancer (DTC) occupies about 80% to 90% of all cases and consists mainly of papillary and follicular carcinomas (29). Total thyroidectomy (TT) and thyroid lobectomy (TL) are the main surgical approaches for DTC, and its most common complications include recurrent laryngeal nerve injury, hypoparathyroidism, hematoma, and wound infection. In this study, as many as 95.79% of patients underwent lesion excision, however postoperatively patients often experience additional psychological burdens, including anxiety and depression. On the one hand, this may be due to the high rate of postoperative complications in patients that undermine their confidence in healing (30), and on the other hand, it is related to fluctuating levels of thyroxine (31) and thyrotropin (32) in the postoperative period. In addition, thyroid autoimmune diseases are inextricably linked to affective disorders (33). In contrast to the overall favorable prognosis, thyroid cancer survivors experience poorer quality of life and a higher risk of suicide. Then, this study is the first to construct a predictive model of suicidal risk factors in thyroid cancer patients and will provide a bridge for oncologists and psychologists to efficiently communicate in order to intervene early in potential suicide attempt and complete suicide.

In the risk factor analysis of this study, age, place of residence, marital status, gender, radiotherapy, race and histologic type were associated with suicide in thyroid cancer patients. We combined just seven easily available and meaningful parameters, and an efficient tool for predicting suicide risk in thyroid cancer patients was built. The nomogram integrates risk factors such as demographic characteristics, socioeconomic status, tumor and treatment characteristics to individualize the prediction of suicide in thyroid cancer patients. We constructed a relatively credible and efficacious suicide prediction model for thyroid cancer using the training cohort. Through internal validation, we confirmed the superior discriminative and calibration capabilities of the model. In addition, the exceptional C-index and decision curve performance contributes to the wide application of the nomogram to a high volume of thyroid cancer samples.

### 4.1 Gender difference

Men are one of the independent risk factors for suicide in thyroid cancer patients, which is consistent with other previously reported cancers (34, 35). As for the general population, three times as many men as women die by suicide in high-income countries, compared with 1.6 times as many in low- and middle-income countries (36). While women are generally considered to be more susceptible to mood disorders and depression, men usually tend to resort to more violent and irreversible ways of contributing to full suicide (37). Secondly, Men have more difficulty regulating mood changes (38) and are more reluctant to seek formal channels of mental health care than women due to stereotypes of traditional masculinity (39). Thyroid cancer is the only nonreproductive cancer with a female predominance, but it is more malignant and more likely to recur in male patients (40), and the despair associated with treatment failure and limited survival expectations makes them more likely to commit suicide (41). In addition, men are at high risk of suicide because they are more susceptible to social isolation that cuts across all spheres of life, including family and society (42, 43).

### 4.2 Age stratification

Advanced age is another independent risk factor for suicide in thyroid cancer patients. Patients <45 years of age have a significantly better prognosis than patients> 45 years of age, even when the degree of cancer aggressiveness is the equal (44). Mortality from thyroid cancer climbs gradually from age 40-45, with recurrence rates peaking from age 60 (45). Individuals with serious illnesses are also more likely to report suicide attempts among those 65 years and older (46). The experience of cancer is one that promotes tremendous personal and spiritual growth (47), and poor physical health and dysfunction also increase suicide risk. Second, older adults who have no income or are not gainfully active are more likely to attempt suicide (37), and the breakdown of relationships with family members and other sources of support contributes in part to suicide in later life (48). Biologically, age-related chronic inflammation may represent a biological substrate linking advanced age to suicide risk (49). In addition, the use of psychotropic medications may be partially involved in the high rates of suicide among older persons, with a much higher number of older persons attempting suicide using tranquilizers and hypnotics than would be expected (50).

### 4.3 The marriage protection effect

With regard to marital status, the risk of suicide was elevated in divorced, separated, windowed, and single (never married) thyroid cancer patients compared to those who were married or cohabiting. Social support, a complex structure containing the important functional and structural parameter of marriage, has traditionally been recognized as having both a direct and buffering effect on the well-being and emotional regulation of cancer patients (51, 52). What’s more, married patients with less lethal cancers, such as thyroid cancer, have higher 5-year survival rates (53). Marriage increases personal fortune as well as pleasurable experiences and, more importantly, prevents suicide by providing social integration and support (54–56). In the 30+ age group, marriage was more protective against suicide in men than in women (57, 58). Similarly, divorce had a significantly greater suicide risk for men than for women (59), which may be due to the fact that women tend to take on the role of caregiver in a marriage and excel in integrating into social networks, while men are more likely to fall into social isolation and loneliness (60). Additionally, suicide in the face of a change in marital status is significantly greater among older people than among younger people, especially in the first year after a marital change (61).

### 4.4 Collective resilience

Studies show a statistically significant difference in suicide rates between racial groups with thyroid cancer, with lower incidence observed in Black patients versus White patients. This, of course, is inextricably linked to the fact that white people are overwhelmingly represented in the SEER database. Compared to other races, black person appear to have lower suicide rates despite social margaretization, racism and colorism, poor access to mental and physical health care, and disproportionately high rates of poverty (62). States with higher proportions of African Americans have lower suicide rates, with interpersonal relationships (i.e., a stronger sense of collectivity in racially dense areas) playing an essential protective role (63). In other words, social support through “racial density” is effective in reducing suicide risk in predominantly African American areas (63–65). Also, strong family ties are key to lowering the risk of suicide among low-income black women (66), and the existence of hope is a significant contributor to the protective factors against suicide among African American (67). Moreover, having a strong and positive racial identity or personal beliefs about being Black has been shown to be protective for women who have attempted suicide (68), and may even confront the negative influence of racism and colorism on suicide (62).

### 4.5 Economic area factor

Our study shows that living in a small urban is considered as one of the independent risk factors for suicide in thyroid cancer patients compared to metropolitan. In fact, the incidence of thyroid cancer is higher in cities than in towns or rural areas in Canada (69). A California-based report claims that rural youth are more likely to have suicide ideation, suicide attempt, even full suicide than urban youth (70). Another Hong Kong-based study has pointed that the strong association between life satisfaction and regional socio-economic characteristics affects suicide (71). Ambient temperature (72), daylight hours (73), firearms support availability (74, 75), socioeconomic status (76), cancer care level (77), and access to mental health care (78, 79) differed by region and were associated with changes in suicide mortality across the United States. From a mathematical or statistical point of view, metropolitan areas, while having the advantage of large sample sizes, inevitably introduce measurement or specification bias. Smaller regions are less susceptible to ecological bias due to less heterogeneity than larger agglomerations (80). However, because small sample sizes lead to highly variable rates, little attention has been focused on suicide-related research in rural-level areas, which may also partially explain the fact that the independent risk factor in this study was small urban rather than the rural area. By the way, more research is needed in the future to further refine the relationship between geographic region type and suicide among thyroid cancer patients, which may help policymakers understand where to focus their improvement efforts.

### 4.6 Treatment denial and the dignity crisis

Refusal of radiotherapy as a risk factor for suicide in patients with thyroid cancer may be related to patients’ desire to maintain autonomy over their lives and loss of confidence in health care. Declining radiotherapy may reflect anticipatory loss of autonomy in end-stage decision-making. Thyroid cancer patients disproportionately value bodily integrity due to visible surgical scars and voice changes, making treatment refusal a potential marker of dignity-related distress (81). A qualitative case study based on the wishes to die of patients with advanced cancer in Switzerland (where assisted suicide is permitted by law) reported that four oncology patients asked to maintain (or regain) control and self-determination over their deaths at the end of their lives (82). They did not want to remain in a state of dependency for a long period of time, fearing that they would not receive appropriate treatment or that they would not be subjected to conventional treatment and non-personalized care in hospitals, nor would they want their doctors to make decisions for them. Other factors such as the fear of being an emotional and financial burden on others, loss of dignity, choice of treatment or meaning in life, and freedom from the confinement of a painful and disfigured body are all possible reasons why oncology patients “long for a self-determined end of life”.

### 4.7 The psychological dilemma of undifferentiated cancer

Our study also found that the histologic type Anaplastic thyroid carcinoma (ATC) increased the risk of suicide death in patients. The phenomenon that histological type of tumor is associated with suicide has been reported in other cancer types such as ovarian cancer (83). The extreme lethality of ATC (median survival <6 months) creates existential distress distinct from other cancers. Patients face rapid functional decline with limited palliative options, amplifying feelings of hopelessness – a well-established suicide precursor (84). Thyroid cancer patients have many unmet psychosocial support needs (85). A serious life-threatening illness may threaten basic expectations of safety, relationships, justice, controllability, certainty, and a long and rewarding life (84). A subgroup of thyroid cancer patients experiencing distress and worry during diagnosis and treatment demonstrated susceptibility to clinical anxiety and depression (86). Although ATC accounts for only 2% of thyroid cancer patients, clinicians should not ignore the exuberant psychological needs of this rare group.

## 5 Advantages and limitations

The main strengths of our study are the large sample of thyroid cancer patients in the United States and the translation of evidence-based risk factors into quantifiable risk factors to help identify high-risk groups and provide transparency and consistency in suicide risk decision-making. Second, our nomogram model balances sensitivity and specificity and provides an overall suicide risk score. Additionally, this model can provide patients with a baseline assessment during contact with a clinician and provide interventions for those predicted to be at high risk for suicide.

There are still some shortcomings in our study. First, psychosocial confounders (e.g., psychiatric diagnoses, substance abuse, childhood trauma) were unavailable in the SEER registry. Future studies integrating electronic health records or patient-reported outcomes may enhance predictive accuracy by capturing these dimensions. Similarly, we did not differentiate between the concepts of suicidal ideation, suicide attempts, and complete suicide when assessing risk factors in patients with thyroid cancer. Second, specific causes of death are often difficult to determine, and ICD-10 cause-of-death codes in the SEER database are unique, so there may be a misclassification bias for causes of death. Third, although the stability and validity of our nomogram have been internally validated, external validation with multiple centers should be performed in the future. Furthermore, the rarity of suicide outcomes (n=169) fundamentally constrained our ability to conduct meaningful subgroup analyses. Finally, the results of this study should be contextualized when generalizing to other countries and regions, as the SEER-17 database originates from a specific region.

## 6 Conclusion

In our study, seven risk factors were screened by combining LASSO and Cox proportional risk regression, and a nomogram was constructed to assess the risk of suicide in thyroid cancer patients. Age at diagnosis, gender, race, marital status, histologic type, radiation therapy, and place of residence were included in the suicide nomogram to quantify the risk of suicide in individual thyroid cancer patients. This nomogram enables early identification of high-risk subgroups who may benefit from targeted psychosocial interventions tailored to thyroid cancer survivorship challenges. Future studies need to be externally validated in multicenter cohorts and should test whether timely individualized interventions can reduce the risk of suicide and further improve mental health and quality of life in patients with thyroid cancer.

## Data Availability

Publicly available datasets were analyzed in this study. This data can be found here: SEER Research Data,17 Registries, Nov 2022 sub (2000-2020)(https://seer.cancer.gov/).
